# Activity of selected enzymes as markers of ecotoxicity in technogenic salinization soils

**DOI:** 10.1007/s11356-019-04830-x

**Published:** 2019-03-20

**Authors:** Joanna Lemanowicz

**Affiliations:** Department of Biogeochemistry and Soil Science, Faculty of Agriculture and Biotechnology, University of Science and Technology, Bernardyńska 6 St., 85-029 Bydgoszcz, Poland

**Keywords:** Enzymes, Enzymatic indices, Salinity, Soda industry, Soil

## Abstract

The activity of enzymes in soil is sensitive to the changes in soil properties affected by biotic and abiotic factors. This study investigates the influence of salinity on some enzymes (catalase CAT, dehydrogenases DEH, alkaline AlP, and acid AcP phosphatase) and pH in 0.01 M CaCl_2_, EC_e_, the content of total organic carbon, and total nitrogen in technogenic salinization soil next to the soda plant. Seven soil sampling sites were selected (S1–S6) in the area close to the soda plant and C (the control). Based on the enzyme activity, also soil indicators were calculated: the resistance index (RS), enzymatic pH indicator $\frac{\mathrm{AlP}}{\mathrm{AcP}}$, the factor of the impact of anthropopressure (IF), the biological index of fertility (BIF), and the indices of biochemical soil activity (BA12 and BA13). The above study did not show one-way changes of the parameters investigated. The relations between the parameters and the activity of catalase, dehydrogenase, alkaline, and acid phosphatase show that they are mostly determined by the state of salinity of the soil environment. The calculated index of resistance (RS), as an effective means of the enzymatic response to environmental stress, facilitated putting the enzymes in the following series: CAT>DEH>AlP>AcP. It shows that catalase and dehydrogenases are most resistant to the anthropogenic factor. The calculated values of BA12 and BA13 indices showed the differences between technogenic salinization soils and the soil sampled from the control. The lowest BIF values were observed at S6 and S3, S4, and C.

## Introduction

Technogenic soils are the soils formed due to the technical and biological reclamation of waste produced due to industrial activity (Uzarowicz 2011; IUSS Working Group WRB 2015). At the same time, the waste usually constitutes a dangerous source of soil environment pollution (Levyk et al. 2007; Blidar et al. 2009; Shestakov et al. 2013). Since the development of mining and other industry sectors leads to an increase in the amount of waste, the reclamation of wasteland becomes a more and more important economic necessity. The problem of the occurrence of such soils has been solved in soil classification systems, e.g., in the world soil resources reference base (IUSS Working Group WRB 2007). The technogenic soils are also formed due to the impact of the soda industry as a result of inadequate storage of the so-called lime sludge in sedimentation tanks (Hulisz and Piernik 2013). Soil salinity is one of the major causes of soil environmental degradation. Soil salinization intensifies due to a high salt concentration, high sodium cation (Na^+^) concentration, and high pH, often due to high CO_3_^2−^ concentration in soil (Daliakopoulos et al. 2016).

The technogenic soil transformation, which reflects the effectiveness of reclamation, can be estimated applying many physical, chemical, and biological methods. Reclamation can alter ecosystem processes that affect soil physicochemical and biological. Study of Xie et al. (2017) showed that reclamation had extremely positive effects on the physicochemical properties and the activities of soil enzymes (dehydrogenase, urease, amylase, acid phosphatase, and alkaline phosphatase) of reclaimed saline soil.

Soil enzymes are biological catalysts, they facilitate the transformation of various forms of energy, and they participate in the processes related to the cycling of bioelements (C, N, P, S). The key sources of soil enzymes are microorganisms, underground plant parts, and soil fauna. The analysis of the activity of soil enzymes provides information on biochemical processes occurring in soil and, as such, they have been studied as soil quality indicators.

The enzymatic activity in soil is regulated by pH and the biomass of microorganisms (Dick et al. 2000; Breza-Boruta et al. 2016), correlated with the organic matter of soil (Bielińska et al. 2013) and moisture content in soil. However, it varies in time and it is limited by substrate availability. Therefore, the role of enzymes for the soil ecosystem is more and more important and it is determined with the relations between soil enzymes and environmental factors (both natural and anthropogenic) which affect their activity. The tests of the enzymatic activity of soil are potential indicators of the quality of ecosystems (Utubo and Tewari 2015; Bayarmaa and Purev 2017; Acosta-Martinez et al. 2018). Frequently, to evaluate the state of the soil environment, the indicators being single physical, chemical, and biochemical parameters, e.g., the content of organic carbon, total nitrogen, C biomass, and the level of FDA hydrolysis are used (Piotrowska-Długosz and Charzyński 2012; Piotrowska-Długosz and Wilczewski 2014), and the content of ATP (Wen et al. 2005), the nitrogen mineralization rate, the activity of catalase, dehydrogenases, phosphatase (Bartkowiak et al. 2017; Riah et al. 2014), urease, and *β*-glucosidase (Adetunji et al. 2017) are used. Soil dehydrogenases (DEH) [E.C.1.1.1] are the major representatives of the oxidoreductase enzymes class. The activity of the dehydrogenases may be considered a good indicator of the oxidative metabolism in soils, and therefore, of microbiological activity (Masciandaro et al. 2001). Catalase (CAT) [EC 1.11.1.6] is an important cellular antioxidant enzyme that defends against oxidative stress and catalyzes the decomposition of hydrogen peroxide to water and oxygen. The enzyme is widely present in nature, which accounts for its diverse activities in soil (Achuba and Peretiemo-Clarke 2008). Catalase activity along side with the dehydrogenase activity is used to give information on the microbial activities in soil. Alkaline (AlP) [EC 3.1.3.1] and acid (AcP) [3.1.3.2] phosphatase catalyze the hydrolysis of organic phosphorus compounds and transform them into an inorganic form of phosphorus, which is then assimilated by plants and microorganisms (Lemanowicz 2018). A growing rate and amount of natural environment pollution has triggered an urgent need of the index-based soil quality evaluation. The soil condition evaluation affected by various natural conditions as well as resulting from the human activity based on a single parameter (e.g., the enzymatic one), or simple indicators, including, e.g., only two parameters, is burdened with some errors. Enzymes are substrate-specific and they are usually related to a single reaction. As such, they cannot reflect the total microbiological activity or the level and direction of transformations of the entire soil metabolism. Similarly, specific chemical compounds present in the soil environment can inhibit or activate the synthesis and the effect of a single enzyme with no effect on the total microbiological soil activity. Considering all the limitations related to the application of single biological parameters or simple indices, evaluating the soil condition, it seems more justifiable to use the indicators developed based on the group of parameters (for example: TOC, clay, pH) reflecting all the essential processes which occur in soil.

We have hypothesized that long-term salinity could affect the activity of the enzymes in soil considerably and could show implications for their resistance.

This study shows the effect of some enzymes on technogenic salinization in soils in the area of the plant of CIECH Soda Polska S.A. The objective has been to explore the dynamics of the activity of four soil enzymes (catalase, dehydrogenases, alkaline, and acid phosphatase) at two soil depths and their responses to changes in soil physicochemical properties (clay, pH, TOC, NT, EC_e_) resulting from long-term salt mining.

## Material and methods

### Description of the study area

The total area of the city of Inowrocław, located at 52° 40′ N; 18° 16′ E in central Poland, is 30.42 km^2^. The climate is moderate cold with a substantial amount of precipitation. The mean annual temperature is around 18.1 °C and the average monthly precipitation is 531 mm. The dominant soil is Mollic Gleysols (in Poland called black earths). According to Hulisz and Piernik (2013), the soils degraded by the technogenically induced salinization process in Inowrocław-Matwy can be classified as Mollic Technosols (Calcaric). In the city center, the Cechsztyn salt dome accumulates. CIECH Soda Polska S.A. produces, e.g., light and heavy soda ash, sodium bicarbonate, calcium chloride, and precipitated calcium carbonate. The production process uses the ammonia Solvay method, which is related to generating a high amount of waste dominated by CaCO_3_ (40%), Ca(OH)_2_ (18%), CaCl_2_ (13%), and NaCl (7%). The waste used to be stored in the so-called sedimentation tanks, without adequate safety measures, which resulted in the penetration of salt to shallow-deposited ground waters, and thus in the salinity of very fertile soils in the adjacent areas. At the same time, the cause of soil salinity related to the impact of that source is the wind spreading around the dried waste from the surface of sedimentation tanks and the emissions of limestone dust during production (Hulisz 2007).

**Fig. 1:**
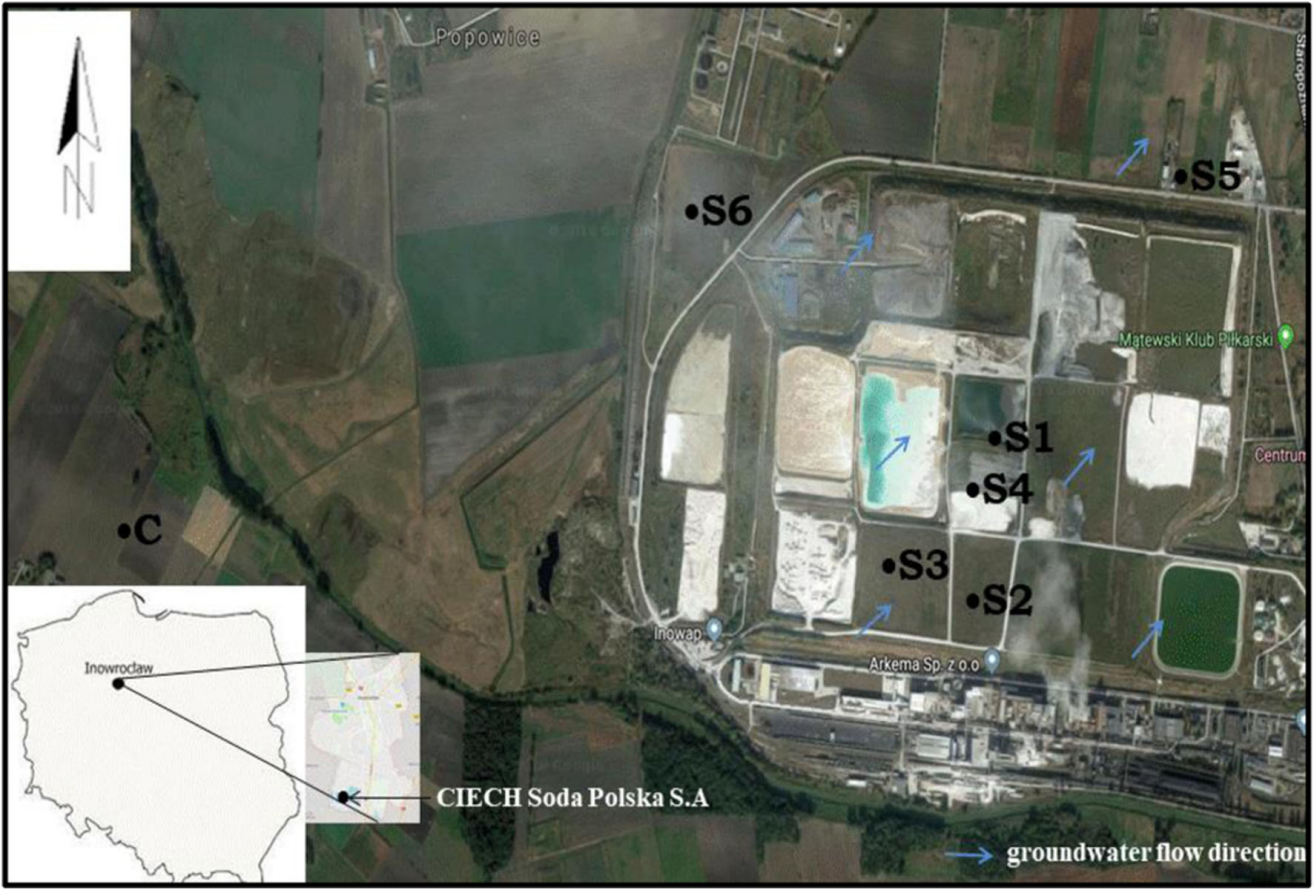
Location of the study area

The soil was sampled from two depths: 0–20 cm and 20–40 cm for the study in spring (April) 2014 from six sites in the area of the soda plant and from the control point (Fig. 1). Site S1 is found around the clarifying-cooling “pond” with a permeable bottom and a drainage system, where carbonates get precipitated as waste. Sites S2 and S3 are the areas where technical and agrotechnical reclamation was completed, while site S4 is the site at the dried pond for ash waters. Site S5 is an agricultural field 500 m away from the soda plant, cropped with winter cereals, while site S6 is the place in the vicinity of the city waste dumping sites, sewage treatment plant, and the soda plant (with numerous communities of halophytes, mainly with *Salicornia europaea*) and site C is the control site.

### Soil physical and chemical properties

In the air-dried soil samples with a disturbed structure, sieved through ø 2-mm mesh sieve, some physicochemical properties were determined: the clay fraction was assayed with the laser diffraction method applying the Masterssizer MS 2000 analyzer, pH in 0.01 M CaCl_2_ measured potentiometrically (ISO 10390), total organic carbon (TOC), and total nitrogen (NT) were determined with the TOC FORMACTS™ analyzer Primacs provided by *Skalar*, electrical conductivity (EC_e_) in soil paste.

### Enzymatic activities and indices

The activity of selected oxidoreductase and hydrolytic enzymes: the activity of dehydrogenases (DEH) [E.C.1.1.1] in soil was assayed with the Thalmann method (Thalmann 1968), the activity of CAT [E.C.1.11.1.6] with the Johnson and Temple method (Johnson and Kl 1964), the activity of AlP [E.C.3.1.3.1] and AcP [E.C.3.1.3.2] phosphatase with the Tabatabai and Bremner method (Tabatabai and Bremner 1969), which facilitated the calculation of enzymatic pH indicator defining the right soil reaction (Dick et al. 2000). Based on the enzymatic activities of the samples, the biological index of fertility (BIF) was calculated according to Stefanica et al. (Stefanic et al. 1984):

1 $$ \frac{\mathrm{AlP}}{\mathrm{AcP}} $$

2 $$ \mathrm{BIF}=\frac{1.5\mathrm{DEH}+100k\mathrm{CAT}}{2}, $$

where *k* is the factor proportionality equal to 0.01.

The indices of biochemical soil activity (BA12 and BA13) (Wyszkowska et al. 2013) were proposed based on the activities of soil enzymes, the content of clay and the content of organic carbon:

3 $$ \mathrm{BA}12={\log}_{10}\mathrm{TOC}\sqrt{\mathrm{DEH}+\mathrm{CAT}+\mathrm{AlP}+\mathrm{AcP}} $$

and

4 $$ \mathrm{BA}13={\log}_{10}\mathrm{Clay}\sqrt{\mathrm{DEH}+\mathrm{CAT}+\mathrm{AlP}+\mathrm{AcP}\ }. $$

The resistance index (RS) determined according to the activity of enzymes to soil was calculated using the formula proposed by Orwin and Wardle (2004):

5 $$ \mathrm{RS}=1-\left[\frac{2\mid D0\mid }{C0+\mid D0\mid}\right] $$

where *D*_0_ = *C*_0_ − *P*_0_, *C*_0_—parameter value in control, P_0_—parameter value in disturbed soil (next to the soda plant). The value of the resistance index is bounded by − 1 and + 1.

The factor of the impact of anthropopressure (IF) on the activity of soil enzymes was calculated according to the formula defined by Borowik et al. (2017):

6 $$ \mathrm{IF}={\frac{P0-C0}{C0}}^{\ast }100, $$

where *C*_0_ and *P*_0_—designations are provided in formula No 5. If IF = 0—no impact, − 1 to 100% inhibition, + 1 to 100% stimulation.

### Statistical analysis

The ANOVA test was performed for the results and analyses were carried out using Statistica 12 for Windows. A two-way analysis of variance was performed to examine the main effect of the method used on soil and the depths on the enzymatic activities and soil physical and chemical properties. The relations between the enzymatic activity and the chemical parameters were estimated using the analysis of correlation based on Pearson’s correlation coefficients (*p* < 0.05). The percentage share of the variability was calculated using *η*^2^ index with the ANOVA variance analysis. Principal component analysis (PCA) was applied using data for soil catalase, dehydrogenase, alkaline and acid phosphatase activities, grain size composition, pH in 0.01 M CaCl_2_, EC_e_, and the content of TOC, TN. The first two principal components (PC1, PC2) were selected for a further interpretation of the results. There was also calculated the coefficient of variation (CV) for the parameters analyzed for the entire study area. As for the values, 0–15%, 16–35%, and > 36% indicate low, moderate, or high variation, respectively (Wilding 1985).

All the analytical measurements were performed with three replications. Arithmetic mean values are shown in tables.

## Results and discussion

### Soil physical and chemical properties

The content of clay fraction (particle size < 0.002 mm) ranged from 4.84 to 8.19% for 0–20 cm and from 4.76 to 9.80% for 20–40 cm. The soils tested showed an alkaline reaction, pH of soil for the depth of 0–20 cm ranged from 7.28 to 7.60 and pH of soil for the depth of 20–40 cm from 7.17 to 7.66 (Table 1).

**Table 1:** The content of clay fraction, pH in 0.01 CaCl_2_ in soil

Sites	Clay [%]		pH CaCl_2_	
	0–20 cm	20–40 cm	0–20 cm	20–40 cm
C	8.19	9.80	7.22	7.32
S1	7.93	7.95	7.45	7.49
S2	7.86	7.17	7.42	7.43
S3	5.64	5.62	7.37	7.50
S4	7.35	5.86	7.64	7.66
S5	4.84	4.76	7.32	7.48
S6	6.62	6.60	7.17	7.28

Salt-affected soils usually exhibit low organic matter contents primarily due to poor plant growth leading to low inputs of organic materials into soil. These soils are also subject to increased losses due to dispersion, erosion, and leaching (Wong et al. 2010). The negative relationship between the content soil organic matter and salinity exhibited in the current work is consistent with Morrissey et al. (2014). The significantly highest content of TOC was found for S1 (the places around the clarifying-cooling “pond,” where carbonates get precipitated as waste) (7.536% in 0–20 cm and 12.480% in 20–40 cm). Saline soils contain carbonates, which complicate the carbon dynamics, and they are also subject to increased losses of organic matter. No significant differences between S2 and S3 were identified (Table 2). TN contents were significantly higher in S6 (0–20 cm) and S3 (20–40 cm) soils, as compared with the other sampling sites.

**Table 2:** The content of organic carbon (Corg), total nitrogen (NT), and electroconductivity (EC_e_) in soil

Sites	TOC [%]		NT [%]		EC_e_ [mS cm^−1^]	
	0–20 cm	20–40 cm	0–20 cm	20–40 cm	0–20 cm	20–40 cm
C	0.289^e^ ± 0.005	0.325^e^ ± 0.004	0.1 96^dB^ ± 0.001	0.201^dA^ ± 0.005	34.7^b^ ± 0.005	85.0^b^ ± 1.306
S1	7.536^a^ ± 0.004	12.48^a^ ± 0.044	0.200^dA^ ± 0.003	0.044^gB^ ± 0.004	19.7^c^ ± 0.458	15.5^f^ ± 0.409
S2	1.361^d^ ± 0.004	0.995^d^ ± 0.034	0.283^cA^ ± 0.004	0.286^cA^ ± 0.004	9.70^d^ ± 0.149	10.5^d^ ± 0.563
S3	1.056^de^ ± 0.004	1.123^d^ ± 0.037	0.329^bB^ ± 0.001	0.397^aA^ ± 0.004	10.3^d^ ± 0.0597	37.9^c^ ± 0.736
S4	6.196^b^ ± 0.004	2.446^c^ ± 0.271	0.082^fB^ ± 0.001	0.130^fA^ ± 0.004	20.6^d^ ± 0.658	20.5^e^ ± 0.755
S5	0.605^e^ ± 0.002	0.464^e^ ± 0.009	0.177^eB^ ± 0.004	0.188^eA^ ± 0.002	35.7^b^ ± 0.620	33.4^d^ ± 0.579
S6	3.922^c^ ± 0.004	3.540^b^ ± 0.115	0.436^aA^ ± 0.002	0.356^bB^ ± 0.003	577^a^ ± 0.183	501^a^ ± 0.805
*η*^2^ depths			0.32			
Sites	88.12		88.79		99.01	

As a result of reclamation, the value of EC_e_ decreased; the significantly lowest values of EC_e_ were recorded in soil S2 and S3 (0–20 cm), 9.70 and 10.3 mS cm^−1^ respectively. The significantly highest values of EC_e_ were in soil S6 (577 mS cm^−1^ in 0–20 cm and 501 mS cm^−1^). Most of the soils according to Jackson’s (1958) classification were very strongly saline (EC_e_ > 16 dS m^−1^). However, no significant difference in EC_e_ across the soil depths was identified. In Polish climatic conditions, the typical salinity level of the soil analyzed was closely linked to the groundwater level. Salinity is greater in regions with lower rainfall. The moisture is evaporated leaving the salts on soil surface and, as a consequence, the EC_e_ values increase. The increase in rainfall can change the EC_e_ and nutrient status of the soil due to leaching. In study of Hulisz and Piernik (2013), Hulisz et al. (2018) presented the salinity characteristics of the soils next to CIECH Soda Polska S.A. in Inowrocław-Mątwy. Values of electrical conductivity (EC_e_) ranged from 43 to 99 dS m^−1^ (indicated strong chemical degradation) (Hulisz and Piernik 2013) and from 15.3 to 122 dS m^−1^ (Hulisz et al. 2018). This variability was correlated with the content of analyzed ions: Na^+^ from 1.83 to 1.9 g dm^−3^; Ca^2+^ from 2.15 to 29.4 g dm–3; Mg^2+^ from 0.01 to 0.05 g dm^−3^; and Cl– from 6.43 to 80.0 g dm^−3^ (Hulisz et al. 2018).

### Enzymatic activities and indices

As shown in Table 3, the soil sampling site clearly inhibits the activity of CAT, DEH, AlP, and AcP. The ANOVA analysis revealed no significant difference in catalase activity at S1, S2, and S3. The significantly lowest CAT activity (0.174 mg H_2_O_2_ kg^−1^ h^−1^ in 0–20 cm and 0.172 mg H_2_O_2_ kg^−1^ h^−1^ in 20–40 cm) was recorded in the soil samples at S6. That soil was identified with the highest value of EC_e_. As reported by other authors (Shi et al. 1994; Telesiński 2012; Bartkowiak et al. 2017), of all the soil enzymes, the most salinity-sensitive are oxidoreductases, especially catalase.

**Table 3:** The activity catalase (CAT), dehydrogenases (DEH), alkaline (AlP), and acid (AcP) phosphatase in soil

Sites	CAT [mg H_2_O_2_ kg^−1^ h^−1^]		DEH [mg TPF kg^−1^ 24 h^−1^]	
	0–20 cm	20–40 cm	0–20 cm	20–40 cm
C	0.208^bA^ ± 0.001	0.200^bcB^ ± 0.001	1.796^dA^ ± 0.008	1.652^cB^ ± 0.007
S1	0.250^aA^ ± 0.033	0.213^bB^ ± 0.001	2.957^bA^ ± 0.033	2.462^bB^ ± 0.036
S2	0.238^aA^ ± 0.009	0.236^abB^ ± 0.004	2.683^cA^ ± 0.010	2.584^bB^ ± 0.046
S3	0.255^aA^ ± 0.003	0.244^aB^ ± 0.004	1.935^dA^ ± 0.05	1.755^cB^ ± 0.028
S4	0.202^bA^ ± 0.001	0.185^cdB^ ± 0.005	1.833^dA^ ± 0.159	1.568^cB^ ± 0.032
S5	0.209^bA^ ± 0.001	0.196^cB^ ± 0.003	3.958^aA^ ± 0.221	3.527^aB^ ± 0.050
S6	0.174^cA^ ± 0.002	0.1 72^dB^ ± 0.003	0.752^eA^ ± 0.007	0.722^dA^ ± 0.006
*η*^2^ depths	5.26		1.17	
Sites	84.21		97.84	
Sites	AlP [mMpNP kg^−1^ h^−1^]		AcP [mMpNP kg^−1^ h^−1^]	
	0–20 cm	20–40 cm	0–20 cm	20–40 cm
C	1.606^cA^ ± 0.003	1.5 91^dB^ ± 0.002	4.657^aA^ ± 0.006	3.027^aB^ ± 0.005
S1	1.5 00^dB^ ± 0.008	1.625^bA^ ± 0.004	2.432^cA^ ± 0.013	1.3 63^dB^ ± 0.006
S2	1.816^bA^ ± 0.003	1.498^cB^ ± 0.007	2.299^dA^ ± 0.011	1.159^eB^ ± 0.008
S3	1.843^aA^ ± 0.006	1.615^cB^ ± 0.004	3.110^bA^ ± 0.007	1.758^bB^ ± 0.011
S4	0.263^fB^ ± 0.006	0.560^eA^ ± 0.006	0.278^fB^ ± 0.005	0.357^gA^ ± 0.004
S5	1.496^eB^ ± 0.004	3.201^aA^ ± 0.007	1.768^eA^ ± 0.011	1.424^cB^ ± 0.005
S6	0.238^gB^ ± 0.005	0.300^fA^ ± 0.003	0.409^gB^ ± 0.002	0.487^fA^ ± 0.004
*η*^2^ depths	2.23		9.93	
Sites	80.87		82.96	

Values followed by the same small letter within each column are not significantly different at *p* < 0.05. Values followed by the same capital letter within each a line are not significantly different at *p* < 0.05. − 0.567; *p* = 0.0345

Different small letters indicate comparison between sites. Different capital letters indicate a comparison among between depths

*η*^2^ [%]; *C*, control

Of the two factors tested, i.e., soil depths and soil sampling sites, the latter was most essential, with the effect on the activity being different for particular enzymes. The soil sampling sites determined, to the greatest extent, the activity of dehydrogenases (*η*^2^ 97.84%), catalase (*η*^2^ 84.21%), acid phosphatase (*η*^2^ 82.96%), and alkaline phosphatase (*η*^2^ 80.87%). The soil depths affected, however much less considerably, the activity of the enzymes. The impact of that factor was highest for acid phosphatase (*η*^2^ 9.93%), catalase (*η*^2^ 5.26%), and alkaline phosphatase (*η*^2^ 2.23%) and lowest for dehydrogenases (*η*^2^ 1.17%). The result study showed that the activities of four enzymes decreased with increasing soil depths, which was consistent with studies Guan et al. (2014) and Lemanowicz and Krzyżaniak (2015). The activity of acid phosphatase was higher than alkaline phosphatase, which coincides with earlier reports (Siddikke et al. Siddikee et al. 2011; Lemanowicz and Bartkowiak 2016) that alkaline phosphatase activity was not predominant in neutral or alkaline soils.

A development of the universal soil fertility index, applicable for all the soils irrespective of their specific nature, is very difficult. Fertility indices should result from varied soil properties, how easy it to measure them, thus they should indicate the directions and changes which occur in the soil environment. According to Gil-Sotres et al. (2005), the enzymatic activity combined with selected chemical properties reflects their fertility and the intensity of the processes which occur in soil.

**Fig. 2:**
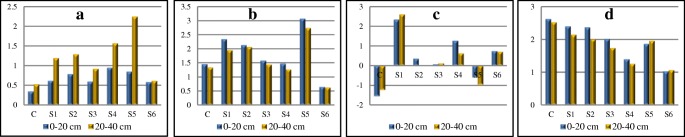
Indices for study enzymes. **a**$\frac{\mathrm{AlP}}{\mathrm{AcP}}$. **b** BIF. **c** BA12. **d** BA13

With the results of the activity of alkaline and acid phosphatase, there was calculated the enzymatic index of soil pH$\frac{\mathrm{AlP}}{\mathrm{AcP}}$. The values of the ratio $\frac{\mathrm{AlP}}{\mathrm{AcP}}$for the soils affected by the soda plant ranged from 0.34 (C 0–20 cm) to 2.25 (S5 20–40 cm) (Fig. 2a). According to Dick et al. (2000), when the value of the enzymatic soil pH index of assumes the value > 0.5, it points to alkaline soil reaction. In most cases, the values exceed 0.50. A higher value was recorded in the soil from the depth of 20–40 cm, which is confirmed by the potentiometric soil pH in 0.01 M CaCl_2_ (Table 1).

The lowest BIF values (Stefanic et al. 1984) were observed at S6 and S3, S4, and C (Fig. 2b). A clearly higher value of the BIF was observed in soil S5 (the agricultural field 500 m away from the soda plant). Saviozzi et al. 2001 observed higher values of BIF in meadow and forest soils as compared to arable fields. The authors thus claim that forest soils show a strong root system and a high amount of organic matter, which makes them considerably different from the soils under agricultural use. It is therefore justifiable to state that the enzymatic activity of soils is sensitive to the soil use.

Using the results of the activity of the soil enzymes, the contents of clay and organic carbon, two indices of biochemical soil activity, BA12 and BA13, were applied (Wyszkowska et al. 2013). It was found that the value of index BA12 was calculated using the content of TOC and the activity of DEH, CAT, AlP, and AcP in soil sampled from the area of the soda plant was higher as compared with the value calculated for the soil sampled from control point C (− 1.55 for 0–20 cm and − 1.24 for 20–40 cm) (Fig. 2c). However, the value of BA13 (calculated based on the content of the clay and the activity of the enzymes studied) was highest in control C (2.626 for 0–20 cm and 2.521 for 20–40 cm) (Fig. 2d). In the soil affected by the soda plant, BA13 was lower and assumed the lowest value at S6 (by about 60% as compared with the control). The calculated values of both indices showed the differences between technogenic salinization soils and the soil sampled from the control. Wyszkowska et al. (2013) found that the activity of those indices depends mostly on the activity of dehydrogenases and the content of carbon.

**Fig. 3:**
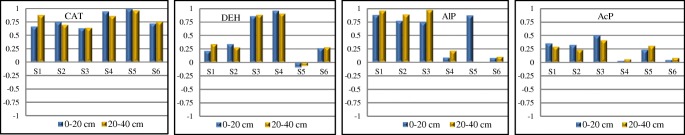
Changes of resistance indices (RS) for catalase (CAT), dehydrogenases (DEH), alkaline phosphatase (AlP), and acid phosphatase (AcP) in soil

According to Kumar et al. (2013), the index of resistance (RS) has an advantage over most of the other indices as it remains bounded even when extreme values are encountered. The resistance was different depending on the enzymes. Catalase showed good resistance and no major differences in resistance were observed in their activities for different sites (Fig. 3). CAT demonstrated the highest resistance (mean 0.798 for 20–40 cm; 0.783 for 0–20 cm) against saline stress followed by AlP (mean 0.571 for 0–20 cm and 0.522 for 20–40 cm) and DEH (mean 0.437 for 20–40 cm; 0.424 for 0–20 cm). Acid phosphatase showed lower resistance to soil salinity (mean 0.249 for 0–20 cm and 0.232 for 20–40 cm). The results Ghollarata and Raiesi (2007) show that the salinity effect on acid phosphatase activity is more pronounced than on alkaline phosphatase activity. This is due to the effects of salinity on plant growth as both microorganisms and higher plants produce AcP in the rhizosphere (AlP in soils is solely derived from microorganisms) (Juma and Tabatabai 1988). In terms of the soil sampling site, index RS for the enzymes studied was different.

**Fig. 4:**
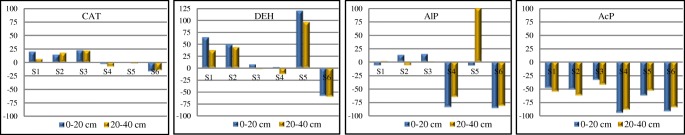
The factor of impact of anthropopressure (IF) [%] of catalase (CAT), dehydrogenases (DEH), alkaline (AlP), and acid (AcP) phosphates activities in soil

Acid phosphatase was the enzyme which was inhibited by salinity. Such an effect was noted in all the sites’ soils (S1–S6). However, the impact of the factor of anthropopressure (IF) showed positive values for catalase (from 6.5 to 22.5%) for S1, S2, and S3 and dehydrogenases (from 37.08 to 120%) for S1, S2, and S5. Also, García and Hernández (1996) stated in their research that the activity of hydrolases (protease, *β*-glucosidase, and phosphatase) was more negatively affected by salinity than that of oxidoreductases (dehydrogenase and catalase). The greatest inhibition of the activity for all enzymes was for soil S6 (Fig. 4).

### Statistical analysis

The relationships between the soil physical-chemical properties and enzymes activities were determined in this study (Table 4). Catalase, dehydrogenases, and alkaline phosphatase activity were negative significantly correlated with salinity (EC_e_), (*r* = − 0.641; *p* = 0.0133), (*r* = − 0.649; *p* = 0.0120), (*r* = − 0.567; *p* = 0.0345) respectively. Also, Frankenberger and Bingham (1982) and Tripathi et al. (2007) stated that dehydrogenase activity was severely inhibited whereas the hydrolases showed a milder degree of inhibition. Such a relationship is proven by the data presented using the PCA method. In the study by Guan et al. (2014), soil EC was also negatively correlated with most of the soil enzyme activities (polyphenol oxidase, *β*-1,4-glucosidase, *β*-D-cellobiosidase, and *β*-xylosidase). The study by Garcia-Gil et al. (2000) showed that soil salinity disperses the clay fraction contained there, and probably the enzymes in our study were less protected and hence the process of their denaturation. While Frankenberger and Bingham (1982) stated that a “salting-out” effect modified the ionic conformation of the active part of the enzyme-protein. According to Tejada et al. (2006), sodium toxicity may alter the active parts of the potential enzymes by salting-out effect. However, Dąbkowska-Naskręt and Bartkowiak (2018) showed that in the soil in the vicinity of the plant CIECH Soda Polska S.A., cations responsible for salinity measured with electrical conductivity were Ca^2+^ and, to a lesser extent, Na^+^. However, other authors’ studies show the stimulatory effect of salts on proteolytic activity (Holik et al. 2017). This is possible thanks to the enzymes that are present in soil, but their activity has not yet been affected by high salinity and alkalinity of the soil environment. According to Wong et al. (2008), soil microorganisms (source of soil enzymes) can adapt to salinity over a long run because salinity causes a change in the structure of microbial populations. The inhibition of enzyme might further reduce the cycling of nutrients and limit crops in these soils (Reitz and Haynes 2003). In the salt-affected soils, there are halophilic and halotolerant microorganisms which can release enzymes under salt stress (Ergasheva and Egamberdieva 2014), thus showing a potential to remedy salt-affected soils (Arora et al. 2014). The research showed that dehydrogenases and alkaline phosphatase were all positively correlated significantly with each other (*r* = 0.693; *p* = 0.0060) (Table 4), indicating that any enzyme activity can reflect other enzyme activities in soil considerably.

**Table 4:** Relationship between selected soil properties

Variables		Regression equation	*r*	*R* ^2^	*p*
CAT	EC_e_	CAT = − 0.000946EC_e_ + 0.223	− 0.641	0.412	0.0133
DEH	EC_e_	DEH = − 0.0032 EC_e_ + 2.483	− 0.649	0.422	0.0120
DEH	AlP	DEH = 0.8094AlP + 1.0488	0.693	0.481	0.0060
AlP	EC_e_	AlP = − 0.0024 EC_e_ + 1.613	− 0.567	0.322	0.0345
NT	EC_e_	NT = 0.0004 EC_e_ + 0.199	0.588	0.346	0.0269

No significant correlations were found between the content of TOC and the activity of the enzymes. A total lack of relationship between the content of TOC and the activity of the enzymes studied in soil could be related with the low participation of humic substances in the total content of organic matter in soils. It limits the availability of easily available carbon which affects the development of microflora producing soil enzymes.

Another measure of the evaluation of the dependence of the activity of enzymes in the soil on some of its properties is the coefficient of determination (*R*^2^) and regression equation. With the value of the coefficient of determination, it was found that 41.2% of the variation in the activity of catalase is due to the variation in EC_e_. The linear regression equations shows that with an increase in EC_e_ by mS cm^−1^, the activity of catalase decreased by 0.000946 mg H_2_O_2_ kg^−1^ h^−1^, dehydrogenases by 0.8094 mg TPF kg^−1^ 24 h^−1^, and alkaline phosphatase by 0.0024 mM pNP kg^−1^ h^−1^ (Table 4). Similarly, the activity of DEH and AlP depended on EC_e_ (42.2% and 32.2%, respectively), while the other 57.8% and 67.8% are accountable for by other soil parameters. Only 34.6% of the NT content was determined by EC_e_.

**Fig. 5:**
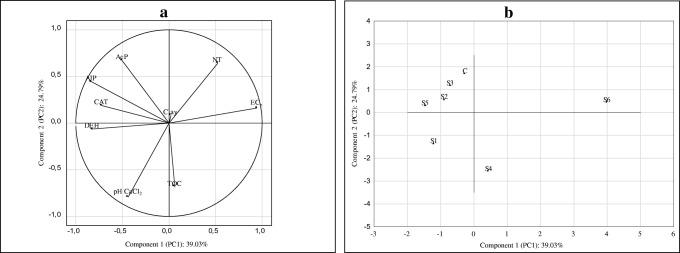
**a** and **b** Component plot in rotated space for studied elements. **a** Plot of variables and **b** loadings graph

To specify the nature and strength of the bonds between the activity of the enzymes studied (CAT, DEH, AlP, AcP) and the content of clay fraction, pH in 0.01 M CaCl_2_, EC_e_, TOC, NT, and environmental variables, the PCA was applied. The resultant diagram (Fig. 5) shows that the two main hypothetical causes of variation (PC1, PC2) accounted for a total of 63.80% of that variation. The first main component conveys 39.03% of information on the soil properties contained in input variables. Most of the variance contained in the first component (PC1) was negatively correlated with the activity of alkaline phosphatase (− 0.835), dehydrogenases (− 0.826), and catalase (− 0.721), however, positively with EC_e_ (0.943) (Table 5). It means that, respectively, 69.7%, 68.2%, 51.5%, and 88.9% of the variance of those variables are accounted for with PC1. This association strongly suggests that these variables have a similar (anthropogenic) source. The distribution of these enzymes is mainly controlled by salinization. The second principal component (PC2) accounts for 24.79% of the data variation. It has shown a negative correlation with pH in CaCl_2_ (− 0.791) and the content of TOC (− 0.685) and positive with acid phosphatase (0.672) (Table 5).

**Table 5:** Values of the three extracted factor loadings (PC1, PC2, PC3) for nine elements

Elements	Component matrix		
	PC1	PC2	PC3
Clay	0.023	0.082	*0.967*
pH CaCl_2_	− 0.437	*− 0.791*	− 0.147
EC_e_	*0.943*	0.162	− 0.083
TOC	0.063	*− 0.685*	0.389
NT	0.524	0.642	− 0.301
CAT	*− 0.721*	0.188	0.056
DEH	*− 0.826*	− 0.063	− 0.319
AlP	*− 0.835*	0.445	− 0.177
AcP	− 0.504	*0.672*	0.485
Variation%	39.03	24.79	17.52

Italic values are statistically significant

Comparing site S6 in Fig. 5b with the principal component forms and factor loadings, it can be concluded that S1 soil was characterized by the highest value of EC_e_ and NT. The soil differed most from the other soils in terms of the properties studied. For this soil, it would be desirable to monitor the physiochemical properties in order to avoid a continued inhibition in biological activity as a consequence of salinity. The next group includes sites C, S2, S3, and S5 with a high activity of CAT, DEH, and AlP. According to Boyrahmadi and Raiesi (2018), at low to moderate salinity levels, the presence of plants may help in stimulating microbial activities, and in alleviating the detrimental influence of salinity on soil enzyme activities.

**Fig. 6:**
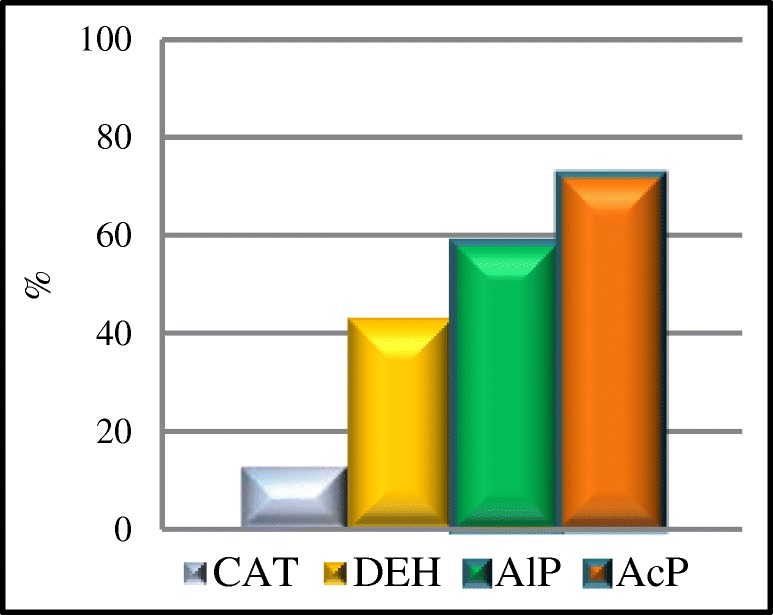
The coefficient of variation (CV%) for catalase (CAT), dehydrogenases (DEH), alkaline phosphatase (AlP), and acid phosphatase (AcP)

There was also calculated the CV for selected soil enzymes exposed to the impact of the soda plant (Fig. 6). The greatest variation in the activity of enzymes in a given area was found for acid phosphatase (72.20%), which points to a high variation in the activity of the enzyme in soil. The enzyme showed the lowest resistance (RS) to soil salinity. The activity of the enzymes analyzed, considering the CV value, were in the order of AcP>AlP>DEH>CAT.

## Conclusion

The above study did not show one-way changes of the parameters investigated. A long-term human impact significantly affected the soil properties under study, which led to a change in the physicochemical properties and the enzymatic activity of soil.

The factors (site, depth) demonstrated a significant effect on the variation of redox and hydrolytic enzymes. However, the location of soil sampling sites and the related intensity of anthropopressure were major factors significantly affecting the enzymatic activity of the soils.

The relations between the parameters and the activity of catalase, dehydrogenase, alkaline, and acid phosphatase show that they are mostly determined by the state of salinity of the soil environment. The factor plays the function of the inhibitor of the enzymes and a wide range of their activity points to their applicability for monitoring the changes caused by human impact.

The factor of the impact of anthropopressure (IF), as compared with the control, showed that acid phosphatase revealed the greatest inhibition, varied depending on the soil sampling site, which can be due to unfavorable changes in physical, chemical, and biological changes in soil as a result of long-term salinization. One can thus assume that the long-term effect of the soda plant is negative for the changes in the activity of hydrolytic enzymes. The least sensitive enzymes, as compared with the control, were oxidoreductases (CAT and DEH).

The calculated index of resistance (RS), as an effective means of the enzymatic response to environmental stress, facilitated putting the enzymes in the following series: CAT>DEH>AlP>AcP. It shows that catalase and dehydrogenases are most resistant to the anthropogenic factor.

In the manuscript, you will find the indices of biochemical activity (BA12 and BA13) which combines the activities of the enzymes and some properties of soil (clay and TOC) and reflects reactions to salinization. The values demonstrate that phosphomonoesterases are the enzymes which are one of the most sensitive indicators of changes in soil pH.

With the PCA, it was found that S2, S3, and S5 are most similar to the control in terms of the parameters studied.

The application of enzymatic indices for a comprehensive evaluation of the ecochemical state of soils around the soda plant facilitates long-term monitoring and identifying the processes which occur in it. It is important that the high enzymatic activity in technogenic salinization soils was observed during the period of two consecutive years, which may indicate that this soil condition has become more stable. The results demonstrate that a long-term follow-up of this research is required.
